# Identification of 2**^nd^chromosome region translocated onto the W chromosome by RFLP with EST-cDNA clones in the Gensei-kouken strains of the mulberry silkworm, *Bombyx mori* L **

**DOI:** 10.1590/S1415-47572009005000105

**Published:** 2010-03-01

**Authors:** Sivaramakurup Sreekumar, Keiko Kadono-Okuda, Ken-ichi Nagayasu, Wajiro Hara

**Affiliations:** Insect Genome Laboratory, National Institute of Agrobiological Sciences, Tsukuba, IbarakiJapan

**Keywords:** sex-limited strains, chromosomal translocation, cDNA markers, restricted fragment length polymorphism, mulberry silkworm

## Abstract

In silkworms, sex-limited strains are either obtained spontaneously or induced by X-rays or gamma rays. When a fragment of an autosome carrying a dominant allele of those genes responsible for certain characters is translocated onto a W chromosome, the female of the successive generations will express these phenotypic characters and sex discrimination can be facilitated. Gensei-kouken strains are sex-limited strains of silkworms developed by irradiating the pupae with gamma rays, by which a portion of the second chromosome is translocated onto the W chromosome. In these improved strains, the females are yellow-blooded and spin yellow cocoons. By using the EST-cDNA clones mapped on the Z chromosome, we identified the sex according to the polymorphic banding pattern or intensity of the signals. Furthermore, by using the clones on the second chromosome, the region of the second chromosome translocated onto the W chromosome was also defined. In both the A95 and A 96 strains selected for the present study, only the mid-portion of the second chromosome was translocated. The differences in length of the fragments translocated in these strains are discussed.

## Introduction

In all the major silk producing countries of the world, the raw silk is being produced from silkworm hybrids. To produce the eggs of the hybrids, it is essential to separate the sexes of the parental breeds before emergence of the moths so as to prevent selfing. The separation of sexes is achieved by sex discrimination at either the larval or pupal stages. Sex separation at the larval stage is not practical on a commercial scale. Though pupal sexing is fairly easy, it is extremely laborious and cumbersome, involving cutting open the cocoons This may damage pupae and cause pest attack by dermestid beetles. These restraints have led to the development of sex-limited or auto-sexing breeds. Identification of sex by sex-limited markings is much simpler, more efficient and minimizes incorrect discrimination. It is well established that the sex chromosome mechanism in the mulberry silkworm *Bombyx mori* is ZZ in the male and ZW in the female, with the W chromosome having a strong female-determining potency (Tanaka, 1916). Furthermore, a number of dominant genes for the manifestation of various characters in the egg, larva, pupa, cocoon and moth stages are located on the autosomes (Fujii *et al.*, 1998). If a dominant gene from the autosome is translocated onto the W chromosome, this gene is expected to be transferred only to female progeny, thus resulting in specific character expression only in females and not in males. Thus, the sexes can be easily separated. This principle was used by Tazima (1941) who succeeded in translocating the dominant sable-marking gene (*p*^*Sa*^) located on the 2^nd^ chromosome onto the W chromosome through X ray irradiation. In the translocated strain, all female larvae possess the sable marking, thereby facilitating separation from male larvae with their normal marking pattern (*+*^*p*^). However, these females were weaker and inferior in cocoon quality than the males due to hyperploidy of the translocated part of the 2^nd^ chromosome, which is large and contains other genes in addition to the *p*^*Sa*^. In order to eliminate these other genes, repeated irradiations were resorted to, whereby the translocated chromosome was made as small as possible. By repeatedly backcrossing this strain to other productive breeds, the sex-limited breed recuperated vigor, almost on a par to that of the normal strains. In 1944, the first sex-limited breed was authorized in Japan for commercial use. Thereafter, several sex-limited breeds have been developed by using genes for larval markings, egg color and cocoon color (Hasimoto, 1948; Tazima *et al.*, 1951; 1955; Sasaki, 1955). Development of sex-limited strains was an industrial boon to sericulture industry, as sex discrimination for the production of silkworm hybrids was facilitated through using the phenotypic characters in the larval or cocoon stages.

Kimura *et al.* (1971) succeeded in developing a sex-limited strain with yellow blood and yellow cocoon-color by translocating the dominant allele of the yellow blood gene ‘*Y*' from the 2^nd^ chromosome onto the W chromosome, through gamma ray irradiation of the mother pupae. This original strain, which carried a T(W;2)*Y* chromosome, facilitated the recognition of females by the yellow blood and yellow cocoons, a result of the linkage of the *Y* gene to the W chromosome. It was named the “yellow cocoon color” strain (Gensei - kouken, in Japanese) (Gen = limited; sei = sex; kou = yellow; ken = cocoon). However due to severe physiological defects, as described by Niino *et al.* (1987), the original strain was further irradiated by Niino *et al.* (1988), giving rise to several strains which differed in their biological characteristics, most probably due to the different structure of the T(W;2) *Y* chromosome.

Setting up the phenotypic trait linkage map of *B. mori* has been under way for many years (Fujii *et al.*, 1998). But DNA markers have an advantage over phenotypic markers through the possibility of producing many markers and distinguishing their polymorphism within the same segregants. The existing RAPD linkage map (Promboon *et al.*, 1995; Yasukochi, 1998) and the AFLP linkage map (Tan *et al.*, 2001) in silkworms are limited to only the strains for which they were constructed and hence such a map cannot be applied to other strains.

Linkage analysis of EST-cDNA clones derived from embryo was carried out by scanning linkage analysis which scans the genotype of each backcross progeny to determine which chromosome was inherited from the heterozygous F1 female (Hara *et al.*, 2001). The reliability of this method has been demonstrated in various populations of segregants (Hara *et al.*, 2002). Kadono-Okuda *et al.* (2002) also applied genetic linkage analysis covering all 28 chromosomes using maternal EST-cDNA clones, and Nguu *et al.* (2005) mapped these EST clones to the respective linkage groups. In this cDNA linkage map based on the segregants in the backcross progeny, 189 cDNA markers were mapped to 28 Restriction Fragment Linkage Groups (RFLGs).

Several cases of spontaneous chromosomal fusion or attachment are reported in *Bombyx mori*, most of which having been identified cytogenetically (Itikawa, 1952; Chikushi, 1959; Sakaida *et al.*, 1996; Doira *et al.*, 1987; Banno *et al.*, 1993; Onimura and Tazima, 1983) and/or by genetic linkage analysis (Tanaka *et al.*, 2000). Neither genetic linkage analysis nor cytological preparation was of help in identifying the attachment region of the autosome to the W chromosome, thus necessitating an alternate strategy. Identification of the fragment translocated to W is of paramount importance, as the excessive part of the fragment, that which otherwise incurs physiological imbalance, could be eliminated by further irradiation. In this context, two sex-limited strains, A95 and A96, wherein the yellow blood gene had been translocated onto the W chromosome, thus resulting in differential expression of cocoon color in both females and males, were selected for the study. Further, it was observed that the survival ratio and hatchability of the female larvae was less than those of male larvae in A96 strain, when compared to A95 strain. This apparently indicated that these two strains may differ as to the length of the translocated portion of the 2^nd^ chromosome. Moreover, Niino *et al.* (1987) showed that the fragment of the 2^nd^ chromosome translocated onto the W chromosome of these sex-limited strains contained the loci of grey egg (*Gr*), yellow blood (*Y*) and inhibitor of lemon (*i-lem*) which have been mapped at the 6.9, 25.6 and 29.5 cM of the 2^nd^ chromosome respectively. Hence for the present investigation, the EST clones mapped onto the 2^nd^ chromosome by Nguu *et al.* (2005) were used to discover the difference, if any, in the length of the translocated fragment of the 2^nd^ chromosome in A95 and A96 strains.

## Materials and Methods

###  Silkworm strains

Gensei-kouken is a group of improved *B. mori* strains in which the females spin yellow cocoons whereas the males spin white ones. A95 and A96 bred by Kimura *et al.* (1971), and maintained separately at Kyushu University, Japan, belong to these Gensei-kouken strains

###  Extraction of genomic DNA

Extraction of DNA was carried out, according to the method described by Hara *et al.* (2001) and Ogoyi *et al.* (2003). Sixteen 3^rd^ instar larvae from each of the A95 and A96 strains were randomly picked from the rearing beds. Subsequently, individual larvae were well-ground in liquid nitrogen. The powder was suspended in 7.5 mL of lysis buffer (50 mM EDTA pH 8.0, 0.5% SDS, containing 100 μg/mL Proteinase K). Samples were incubated at 50 °C for an hour with occasional swirling. The incubated samples were extracted with equal volume of TE (10 mM Tris HCl and 1 mM EDTA, pH 8.0)-saturated phenol in a rotary for 20 min and centrifuged (4000 g for 10 min). From the aqueous phase genomic DNA was precipitated using 1/10^th^ volume of 3 M sodium acetate and 2.5 times absolute ethanol. Precipitated DNA was wound onto a Pasteur pipette with a curved tip and dissolved in 15 mL of TE buffer in a rotor, with occasional gentle mixing until complete dissolution. A certain volume was concentrated to 2.5 mL. by 2-butanol extraction. Butanol was removed by 3-fold extraction with diethyl ether and traces of ether being removed by evaporation at 45 °C for half an hour. Solid cesium chloride (2.5 g) was gradually dissolved in the DNA solution and ethidium bromide (10 mg/mL) added, whereupon all was gently mixed. Samples were kept in the dark for 1 h and then ultra-centrifuged (450,000 x g for 3 h at 25 °C) in a Beckman NVT100 vertical rotor. Isolated DNA samples were extensively dialyzed in TE buffer and DNA concentration adjusted to 1 μg/5 μL.

###  Southern Blot hybridization

Southern blot hybridization was as described by Hara *et al.* (2001). Genomic DNA (5 μg) from each of the 16 samples of both the A95 and A 96 strains were digested separately overnight with various restriction enzymes, viz. *Bam*HI, *Eco*RI, *Hind*III, *Kpn*I and *Sac*I, in a total reaction mixture of 200 μL at 37 °C. The digested DNA was ethanol precipitated in the presence of tRNA and reconstituted in TE and loading sample buffer. 1 μg of digested DNA was used for agarose gel electrophoresis (1%) in TPE buffer (90 mM Tris phosphate, 2 mM EDTA, pH 8.0). 0.25 N HCl was used for depurination (15 min) followed by denaturation in 1.5 M NaCl and 0.5 M NaOH for 30 min at room temperature. DNA was vacuum transferred to Nylon membranes using 20x SSC (0. 015 M NaCl, 0.015 M sodium citrate). Membranes were exposed to UV for 2-3 min for cross-linking. Cross-linked membranes were baked at 120 °C for 30 min. Prehybridization was done in a solution of 50% deionized formamide, 5x SSC, 0.02% SDS and 0.5% blocking reagent at 42 °C for 1 h in a Seal-a-Meal bag immersed in a water bath with constant shaking. DIG labelled RNA probes pertaining to Z and the second chromosome were prepared as described by Hara *et al.* (2001). Hybridization was carried out overnight with respective probes in the same prehybridization buffer at 42 °C. Approximately 0.1-0.25 μg of probe per membrane (6 cm x 11 cm) in 4 mL buffer were used. Membranes were washed twice in 2x SSC, 0.1% SDS at room temperature for 20 min, followed by two high stringency washes at 65 °C in 0.1x SSC, 0.1% SDS at 65 °C.

Membranes were incubated with anti-DIG alkaline phosphatase antibody (Boehringer Mannheim) for 30 min, and unbound antibody was removed by washing with wash buffer (0.1 M maleic acid, 0.15 M NaCl pH 7.5 containing 0.3% Tween 20) at room temperature. Membranes were soaked in detection buffer (0.1 M Tris HCl, 0.1 M NaCl, pH 9.5) for 5 min, before reacting with CSPD substrate (Boehringer Mannheim) for 5 min in sealed bags. The substrate solution was drained off from the membrane by gently squeezing the bags, and the membrane was then exposed to X-ray film (Kodak X-Omat, USA) for 2 h. Membranes were rehybridized after removing the previous probe by washing the membrane in reprobing solution (0.2 M NaOH, 0.1% SDS) for 30 min at 37 °C with gentle agitation, followed by washing with distilled water several times to remove traces of NaOH, and once with 2x SSC.

###  cDNA markers

cDNA markers pertaining to the Z- chromosome and the second chromosome used in the study were derived from previously prepared maternal (m-series, Kadono-Okuda *et al.*, 2002) or embryonic cDNA libraries (e-series, Hara *et al.*, 2001). Clones mapped on the Z- chromosomes (e16, m289, m225, m1004, m132, m261 and m47) were used for comparing the hybridization pattern in both males and females. Clones specific to the second chromosome (m202, m96, e61, m113, m194 and e41) were used in identifying the translocated chromosome on the W chromosome (Table 1).

## Results

With the goal of determining the length of the second chromosome translocated onto the W chromosome in the sex-limited strains of mulberry silkworms (A95 and A96), we used the Z chromosome specific probes for identification of sex and second chromosome specific probes for the identification of the translocated fragment.

###  Identification of sex by Z chromosome specific cDNA clones

**Figure 1:**
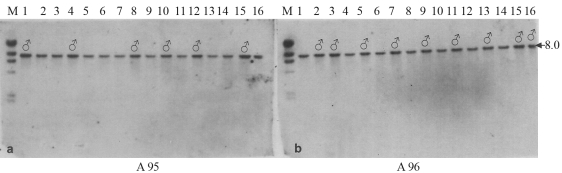
Hybridization pattern of the A95 (a) and A96 (b) strains of silkworm with the Z chromosome specific probe, m 1004. M = marker. N. 1 to 16 indicates genomic DNA of 16 individuals from each strain, digested with *Kpn*1, in serial order. Note the two copy signal intensities representing the two Z chromosomes in the males and the single copy- signal intensity representing the one Z chromosome in the others, as expected for females.

The DNA from randomly selected 3^rd^ instar larvae of both the A95 and A96 strains was digested with restriction enzymes and probed with Z chromosome specific probes (Table 1). It should be mentioned at this point, that the sex chromosome mechanism in silkworms is of the ZZ : ZW type, with two Z chromosomes in the male and one Z and one W chromosome in the female. The hybridization pattern of genomic DNAs of A95 and A96 strains digested with *Kpn*I and probed with the m1004 clone are indicated in Figures 1a and 1b respectively. Samples 1, 4, 8, 10, 12 and 15 of the A95 and 2, 3, 5, 7, 9, 11, 13, 15 and 16 of the A96 strains all showed a single band 8.0 kb in size with two copy-signal intensities representing the two Z chromosomes in all these samples, thus clearly indicating that these could be males. The remaining samples (2, 3, 5, 6, 7, 9, 11, 13, 14 and 16 of A95 and 1, 4, 6, 8, 10, 12, and 14 of A96) had a single band of 8.0 kb showing single copy-signal intensities, thus representing one Z chromosome as expected for females. With other Z-linked probes, as e16, m289, m132, m225, m261 and m47, the same banding pattern with double intensities were obtained as in case of the m1004 probe. Thus, the samples which showed the bands with two copy signal intensities were identified as males. Having identified the males by using Z chromosome specific probes, we loaded the DNA of females on the left and those of males on the right side of the agarose gel in the successive experiments.

###  Identification of the 2^nd^ chromosomal fragment translocated onto the W chromosome

**Figure 2:**
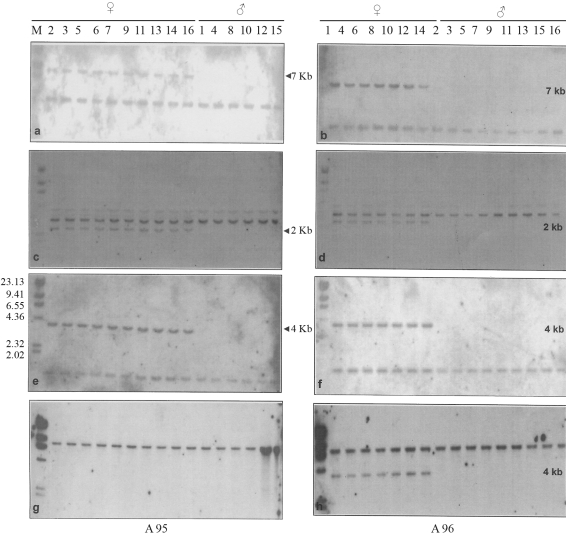
Hybridization pattern of DNA from the A95 and A96 silkworm strains, digested with *Kpn*1, with second chromosome specific probe. a,b:m96; c,d:e61; e,f:m113; g,h:m194 [M, marker, arrow indicates the extra band specific to females].

When m96, which had already been mapped (Table 1) on the 2^nd^ chromosome, was used as a probe for hybridisation with the DNA of males and females of both strains digested with *Kpn*1, these shared a single common band with a uniform intensity of 4.0 kb. However, there was an extra band of 7.0 kb in all females from both strains (Figure 2a,b). In the case of the e61 probe, both males and females of the two strains (A95 and A96) showed two bands, one of 4.0 kb and the other 3.0 kb common to both, besides an extra band of 2.0 kb, observed only in females (Figure 2c,d). When m113 was used as a probe (Figure 2e,f), a common band of 1.5 kb was present in males and females of both strains. Even so, an additional band (4.0 kb) was found only in females.

Interestingly, when m194 was used as a probe for hybridization (Figure 2g), the A95 strain showed only one monomorphic band of 8.0 kb in both sexes, with no extra band being observed in females. On the contrary, in the A96 strain (Figure 2h), a common band of 8.0 kb was found in both sexes, while the females showed an additional band of 4.0 kb.

**Figure 3:**
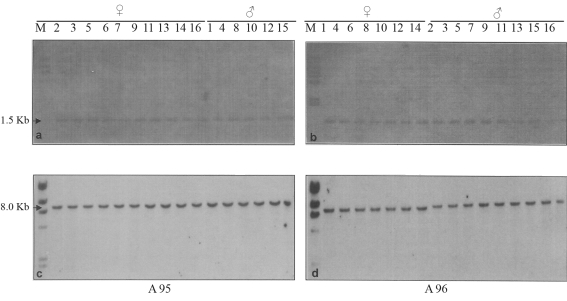
Hybridization pattern of DNA from the A95 and A96 silkworm strains, digested with *Kpn*1, with distal second chromosome specific probe, m202 and e41. a,b: m202; c,d:e41 [M, marker, arrows indicate common bands observed in both sexes].

**Figure 4:**
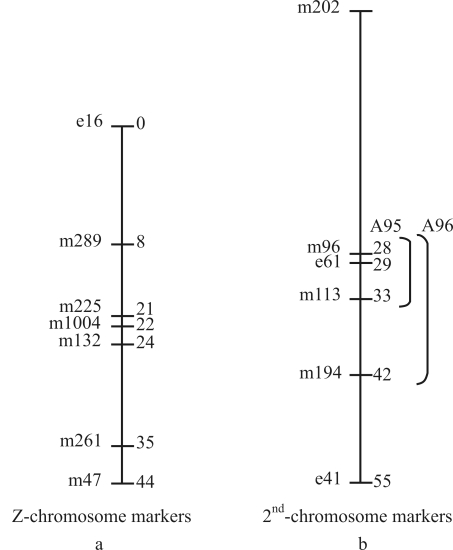
Diagrammatic representation of the linkage map of Z (a) and second (b) chromosomes with the EST markers considered for this study. The numbers on the right represent distances in cM between the clones, and on the left indicate the EST-cDNA clones used.

When the clones, m202 and e41 were used as probes, all the individuals of both A95 and A96 strains showed a single band without any polymorphism (Figure 3a-d). The bands had a size of 1.5 kb in both strains with the m202 probe, whereas it was 8.0 kb size in both when e41 was used as the probe. It is worthy of note that the m202 (0.0 cM) and e41 (55 cM) clones were located at the opposite ends of the second chromosome (Figure 4b).

## Discussion

Our aim was to identify the portion of the second chromosome which was translocated onto the W chromosome in the Gensei-kouken strains (A95 and A96). Z chromosome specific probes for both identification itself and confirmation of sex, and second chromosome specific probes for the identification of the translocated fragment, were used in the present study. To our knowledge, this is the first report on the identification of the chromosomal fragment translocated onto the W chromosome by using the RFLP of EST-cDNA clones.

Our results are primarily based on an analysis of differences in dosage (signal) intensities and the appearance of additional bands of RFLPs among individuals when hybridized with various clones (Table 1) mapped on the first (Z) and second linkage group, and for which high quality DNA is essential. The inclusion of ultracentrifugation in the final step of DNA extraction was helpful in obtaining highly purified DNA, free from RNA and other contaminants, thus aiding in identifying dosage intensities in hybridization signals when probed with different EST-cDNA clones.

Genetically, RFLP is a co-dominant marker. The requirement of a large quantity of DNA was a major constraint for its use as an effective tool in the analysis of polymorphism. However, using ultracentrifugation of DNA with cesium chloride and with modified techniques for Southern blot hybridization, we were able to reduce the quantity of DNA to a minimum of 1 μg per well in agarose gel, and reuse the filter several times (up to 20) after reprobing the filters with NaOH. It is worth mentioning that in the previously reported Southern blot hybridizations (Shi *et al.*, 1995; Mills and Goldsmith, 2000), the amount of DNA used was ten times that of the present study.

As the chromosome set-up in silkworms is ZZ for males and ZW for females, Z chromosome-specific probes were used for identifying sex based on copy signal intensity. Thus, six individuals (out of the 16 randomly selected larvae) of the A95 strain with two copy-signal intensities were identified as males (Figure 1). Likewise, and based on the banding pattern, nine individuals were identified as males from the A96 strain group. All Z chromosome-specific probes yielded two copy-signal intensities, irrespective of the restriction-digestion enzymes used. Hence it became clear that all the clones belonging to the sex chromosomes could be used for the identification of sex.

The appearance of an extra band exclusive to females when using second chromosome specific probes, *viz*. m96, e61, m113 and m194, was owing to the fragment of the second chromosome corresponding to the specific region translocated onto the W chromosome. In the A95 strain, there was an additional band in females with all the probes, except m194. The monomorphic band shown by both males and females of the A 95 strain when probed with m194 showed uniform intensity, indicating the absence of this region in the translocated 2^nd^ chromosome. As to the A96 strain, all the four 2^nd^ chromosome specific probes, including m194, generated an additional band (Figure 2h), thus indicating that the region of the second chromosome from m96 (28 cM to m194 (42 cM), including the regions representing e61 (29 cM) and m113 (33 cM), are translocated (Figure 4b). The difference noted between these two strains was that in the A95 strain, the translocated 2^nd^ chromosome fragment included within the regions from m96 (28 cM) to m113 (33 cM) was translocated, whereas in the A96 strain, the fragment including regions from m96 to m194 (42 cM) was translocated, that is, a considerably longer fragment was translocated in A96 when compared to the A95 strain (Figure 4b). The absence of an additional band observed among the individuals in both strains when hybridized with clones at the proximal end represented by m202 (0.0 cM) and the distal end covering the clone e41 (55 cM), indicated that fragments corresponding to the opposite ends were not included in the fragment translocated onto the W chromosome (Figure 3 a-d).

Females with W; 2 translocation are hyperploid with three doses of genes for the *Y* locus, the original pair of 2^nd^ chromosome carrying the two wild type alleles (+ +) for white blood, and the translocated part of the 2^nd^ chromosome the yellow blood gene *Y* ,whereby the genetic constitution of the female is + + *Y.* On the other hand, males carry only the two wild types alleles (+ +). As the wild type (+) and Yellow blood (*Y*) are different alleles and the restriction sites might be different, this would result in a polymorphic banding pattern. The results clearly showed additional bands in females (Figure 2), thereby substantiating the above.

The yellow color of the cocoons spun by the females in these Gensei-kouken strains was due to the yellow blood gene (*Y*) located on the second chromosome mapped at 25.6 cM, together with the cocoon color gene (*C*) on the 12^th^ chromosome mapped at 7.2 cM (Kimura *et al.*, 1971). Based on hybridization of genomic DNA in the A95 and A96 strains by using second chromosome-specific clones, it could be presumed that the yellow gene *Y* might be located in a region between m96, which is mapped at 28 cM, and m202 (0.0 cM), which is at the proximal end of the 2^nd^ chromosome. It may be mentioned here that we did not include for analysis, the clones marked between m202 (0.0 cM) and m96 (28 cM), as well as those between m194 (42 cM) and e41 (55 cM) in the second linkage group. There was a clear indication of the translocation of the 2^nd^ chromosomal fragment in the m96 region (28 cM) in both the A95 and A96 strains. Furthermore, as the Y gene, which is mapped at 25.6 cM, is present in both strains, the translocated fragment extends beyond m96 (28 cM), includes the *Y* gene (25.6 cM), and proceeds at least up to the Grey egg gene (*Gr*) which has been mapped at 6.9 cM. Since m202 located at the proximal end (0.0 cM) of the 2^nd^ chromosome did not show an additional band in the two strains, it is clear that the translocated fragment does not include the proximal portion in neither. Hence the translocated region could be > m96 but < m202. Niino *et al.* (1987) through backcross breeding experiments concluded that the fragment of the 2^nd^ chromosome translocated onto the W chromosome of the sex-limited yellow cocoon strains contained the loci of yellow blood (*Y*), this having been mapped at 25.6 cM, and which clearly supports our assumption that the region of translocation cannot be restricted up to the m96 probe in the strains we studied. Furthermore, in the A95 strain, the detection of additional bands in m96, e61 and m113 clones, and their absence in the m194 clone have amply proved that the translocated fragment is at least up to 33 cM. However, we are not sure about of its further extension, as there were no markers between 33 cM and 42 cM. Hence the possibility of the translocated region being greater than 33 cM and smaller than 42 cM cannot be ruled out in the A95 strain. On the contrary, a fragment up to 42 cM was found to be translocated in the A96 strain, which showed extra bands with m96, e61, m113 and m194. But, there was no additional band when hybridized with e41 (55 cM) which is mapped at the distal end of the 2^nd^ chromosome. Hence, the data clearly indicate that the translocated fragment in the A96 strain is at least up to 42 cM. However the length of the fragment translocated cannot be restricted only to this point, as there were no markers between 42 cM and 55 cM.

The present study has clearly shown that the length of the translocated fragment of the 2^nd^ chromosome differs between A95 and A96 strains. It is longer in the latter, reaching at least 42 cM, and spans the markers from m96 to m194, whereas in A95 it is shorter, extending 33 cM, flanking the regions between m96 and m113. On considering the length of the translocated chromosome to be longer in A96, this additional part could probably be held responsible for the higher mortality rate in A96 females than in A95 (Hara and Nagayasu, personal communication). Apart from this minor difference in translocated fragment length, no further marked disparity could be found to induce us to believe that both these strains bred by Kimura *et al.* (1971) were very closely related and had evolved from the same genetic stock. Close relationship between these strains was further confirmed by the identical Southern hybridization pattern as seen in Figures 1a and b, with two copy-signal intensities in males and a single copy signal intensity in females., thereby implying the possibility of the same origin of Z chromosomes in both strains.

Chromosomal breakage due to either spontaneous or induced translocation, or to deletion is common among silkworm stocks (Doira *et al.*, 1987). The sex-limited *p*^*Sa*^ sable marking strain had a translocation between the W and the second chromosome, whereas the normal marking strain (+^*P*^) had evolved by eliminating the excess part of the translocated second chromosome (Tazima, 1944). Tanaka *et al.* (2000) found that the sex limited *Pb* silkworm strain had 27 chromosome pairs whereas a normal strain had 28 bivalents. By backcross-breeding, it was found that the large chromosome was composed of a W chromosome to which portions of the second and fifth chromosome had been translocated. Spontaneous chromosomal attachments like the 6^th^ and 14^th^ chromosomes (Itikawa, 1952; Chikushi, 1959), the 6^th^ and 7^th^ chromosomes (Sakaida *et al.*, 1996), the 23^rd^ and 25^th^ chromosomes (Doira *et al.*, 1987; Banno *et al.*, 1993), and W and the 5^th^ chromosomes (Onimura and Tazima, 1983), have also been reported in *B. mori*, most of which based on cytological observations. In *Ephestia kuehniella* (Lepidoptera), the fusion of an autosome with the W chromosome has been reported (Traut, 1986). In *Ephestia,* several other structural mutants of the W chromosome were induced by irradiation such as a T(A;W) translocation by Traut *et al.* (1986), or four T(W;Z) translocations by Marec and Traut (1994).

Our findings indicate the possible existence of breakage sites in the second chromosome in these two strains. Probably, the second chromosome of the A95 strain had broken at two points - one near the Grey egg gene (*Gr)* in the 6.9 cM region and the other near the m113 region. Likewise, there were breaks in the A96 s chromosome at two points - one near the Grey egg gene (*Gr)* also in the 6.9 cM region and the other near the m194 marker region. Fujiwara and Maekawa (1994) reported a breakage site near the proximal end of the second chromosome in *B. mori*, and demonstrated the independent existence of a fragment of the second chromosome in the mottled stripped (*p*^*Sm*^) of *B. mori* by using RFLP. Our observations suggest that the broken fragment of the second chromosome does not exist as an independent entity, but becomes translocated to the W chromosome. Absence of crossing over in the females (Sturtevant, 1915) leads to the continued inheritance of this fragment in successive generations. Since the second chromosome has broken at two points in these strains before translocation onto the W chromosome with one end, it is obvious that the other end of the broken chromosome might have lost the telomeric sequence which may generate unstable conditions for its long-term maintenance. This end might have been restored by the addition of a telomeric sequence from the W chromosome in order to re-establish stability in the translocated fragment. Fujiwara *et al.* (2000) showed that a small chromosome fragment, the *Ze* fragment, responsible for a mosaic pattern in the mottled mosaic strains of the silkworm, has the telomeric repeats only at one end, and is even inherited without the telomere. Fujiwara and Maekawa (1994) showed that the broken ends of chromosomal fragments generated due to X-ray irradiation could be basically restored by *de novo* addition of telomeric repeats, and the structural difference of teleomeres may be related to the stability of chromosomal fragments. They used FISH with the *Bombyx* telomeric sequence (TTAGG)_n_ as a probe to study their possible role in restoring the broken end of the second chromosome fragment in mottled (*p*^*m*^) strains. Possibly this is also the case in sex-limited strains. Interstitial telomeric sequences (ITS) can serve as templates for formatting a new functional telomere in Lepidoptera (Rego and Marec, 2003).

Abe *et al.* (1998) identified three W-specific RAPD markers. Subsequently, nine more W-specific RAPDs were detected (Abe *et al.* 2005a) and thus a total of 12 W-specific RAPDs were identified. Further, it was observed that retrotransposable elements are the main structural components of the W chromosome (Abe *et al.* 2005b). Recently, Abe *et al.* (2008) have shown the presence or absence of the 12 W-specific RAPDs in three sex-limited yellow cocoon strains. The original W chromosome of the C125 strain had all the 12 W-specific RAPD markers whereas in the T(W;2)*Y-* Chu type strain there were six and in the *T(W;2)Y-* Abe and - Ban type had only one of the 12 W specific RAPD markers. However, since these recently published works were not available when we conducted the study, these W specific markers could not be used. Yoshido *et al.* (2005) identified individual *Bombyx* chromosomes by using BAC clones as FISH probes. Further studies with FISH make it possible to locate the translocated fragment on the W chromosome in these strains, as shown by Sahara *et al.* (2003). Nevertheless, in the present study, by using the RFLP of the EST cDNA clones mapped on the molecular map developed by Nguu *et al.* (2005), we were able to identify simultaneously both the translocated fragment of the second chromosome on the W chromosome and the differential length of the translocated fragment of the chromosome in the two Gensei-kouken strains bred.

## Figures and Tables

**Table 1:** cDNA clones (markers) used in the present study.

cDNA library of *B.mori*	cDNA clones specific to				
	Z chromosome			2^nd^ chromosome (Autosome)	
	Clone n.	Locus		Clone n.	Locus
e- (Embryonic) series	E 16	0.0		e41	55.0
				e61	29.0

m- (Maternal) series	M289	8.0		m202	0.0
	m225	21.0		m96	28.0
	m1004	22.0		m113	33.0
	m132	24.0		m194	42.0
	m261	35.0			
	m47	44.0			
